# Development and Characterization of Drug Loaded PVA/PCL Fibres for Wound Dressing Applications

**DOI:** 10.3390/polym15061355

**Published:** 2023-03-08

**Authors:** Ali Afzal, Mohammed Jalalah, Abid Noor, Zubair Khaliq, Muhammad Bilal Qadir, Rashid Masood, Ahsan Nazir, Sheraz Ahmad, Faheem Ahmad, Muhammad Irfan, Munazza Afzal, Mohd Faisal, Saeed A. Alsareii, Farid A. Harraz

**Affiliations:** 1 Department of Textile Engineering, School of Engineering & Technology, National Textile University, Faisalabad 37610, Pakistan; 2 Promising Centre for Sensors and Electronic Devices (PCSED), Advanced Materials and Nano-Research Centre, Najran University, Najran 11001, Saudi Arabia; 3 Department of Electrical Engineering, College of Engineering, Najran University, Najran 11001, Saudi Arabia; 4 Department of Textile Technology, School of Engineering & Technology, National Textile University, Faisalabad 37610, Pakistan; 5 Department of Materials, School of Engineering & Technology, National Textile University, Faisalabad 37610, Pakistan; 6 Institute of Microbiology, University of Veterinary and Animal Sciences, Lahore 54000, Pakistan; 7 Department of Chemistry, Faculty of Science and Arts, Najran University, Najran 11001, Saudi Arabia; 8 Department of Surgery, College of Medicine, Najran University, Najran 11001, Saudi Arabia; 9 Department of Chemistry, Faculty of Science and Arts at Sharurah, Najran University, Sharurah 68342, Saudi Arabia

**Keywords:** antimicrobial, drug release, medical, polycaprolactone, wound dressing

## Abstract

Nowadays, synthetic polymers are used in medical applications due to their special biodegradable, biocompatible, hydrophilic, and non-toxic properties. The materials, which can be used for wound dressing fabrication with controlled drug release profile, are the need of the time. The main aim of this study was to develop and characterize polyvinyl alcohol/polycaprolactone (PVA/PCL) fibres containing a model drug. A dope solution comprising PVA/PCL with the drug was extruded into a coagulation bath and became solidified. The developed PVA/PCL fibres were then rinsed and dried. These fibres were tested for Fourier transform infrared spectroscopy, linear density, topographic analysis, tensile properties, liquid absorption, swelling behaviour, degradation, antimicrobial activity, and drug release profile for improved and better healing of the wound. From the results, it was concluded that PVA/PCL fibres containing a model drug can be produced by using the wet spinning technique and have respectable tensile properties; adequate liquid absorption, swelling %, and degradation %; and good antimicrobial activity with the controlled drug release profile of the model drug for wound dressing applications.

## 1. Introduction

Skin is the human body’s largest organ and has the functions of regulating fluid flow, body temperature, and enable sensation (e.g., cold, heat, pain, and touch). Skin also provides a semipermeable protective layer against pathogens [1]. In a chronic wound, tissue homeostasis is interrupted [2], while vasculature is partially damaged or fully damaged in the case of an acute wound, limiting the regeneration of cells [3]. The period to close the wound is associated with an increased risk of complications such as pain, infection, and the occurrence of scarring [4]. Wound infection is, therefore, the main reason for morbidity, mortality, and delay in wound healing [5,6]. More than 11 million people worldwide are affected by burn injuries annually. In burn units, the prevalence of infections is about 66% [7,8].

There are several wound healing dressing products available to improve healing. The choice of material for a particular type of wound is therefore essential for proper healing [9]. Additionally, to operate, the dressings must be in contact with the wound [10,11]. Dressings also prevent trauma, reduce wound infection, keep matrix materials in contact with the wound, and improve the wound’s electrical gradient [6,12].

Synthetic polymers are being used in different areas including drug delivery applications [13,14]. In biodegradable polymers, PVA is one of the most used polymers and has a lot of good properties that make it suitable to use in medical applications, especially in wound healing properties in both in vivo and in vitro studies [15,16]. PVA has been reported to be used in tissue engineering, wound dressings, and ophthalmic applications [17,18]. PVA hydrogels have also been used in the drug delivery system due to their similarity with natural tissues and excellent biocompatibility [19,20,21].

PCL is another biodegradable polymer that has many applications in biomedical applications. PCL has been used in tissue engineering, drug delivery, and wound dressing [22,23,24,25,26,27]. Mehteroglu et. al. prepared and characterized electrospun PCL fibres as a potential dressing for wound healing applications [28]. PCL shows its exceptional properties when blending with different natural polymers. Prolonged biodegradation and hydrophobicity of PCL were modified by blending it with collagen, hyaluronic acid, gelatine, chitosan, etc. [29,30,31]. Drug delivery vehicles of different sizes (micro and nano) based on PCL are undergoing extensive research [32,33].

To develop fibres from different polymers, various techniques including wet spinning, gel spinning, melt spinning, and, most widely, electrospinning have been used to produce nanofibers [34,35]. Among these techniques, wet spinning is one of the most used techniques to develop medical fibres [36]. By using wet spinning technique, fibres of PVA [37], PCL [38], chitosan and alginate [39,40,41], psyllium husk [42,43], cellulose [44], polylactic acid [45], silk fibroin [46], polypyrrole [47], and collagen [48,49] have been prepared. Due to its low cost, scalability, and reproducibility, as well as the fact that it is a safe and clean technique, interest in using the wet spinning technique for pharmaceutical and biomedical applications has increased [50].

From previous studies, it was revealed that PVA has been used in the medical and textile industry due to its non-toxicity, biomedical compatibility, biodegradability, aqueous solubility, chemical resistance, and low environmental impact [51]. Like PVA, PCL is also one of the most important used polymers in medical applications for drug delivery, sutures, and tissue engineering [52,53]. From the literature review, it was found that both PVA and PCL have been used in many applications, but no significant data were found on the development of PVA and PCL fibres as a blend containing the drug using any scalable production process. The PVA/PCL hybrid fibre mats loaded with drugs are reported in the literature developed by the electrospinning process [53,54,55,56,57,58]. Such electrospun materials cannot be scalable for commercial production due to the low production rate and cost. Composite structures developed with electrospun PCL fibres with PVA hydrogels as hybrid composite structures are also reported [59]. A fibre having hydrophilic and hydrophobic parts will provide the best combination for the transdermal drug delivery system. Therefore, in this study, PVA/PCL fibres containing the drug were developed by using the wet spinning technique for wound dressing applications.

## 2. Materials and Methods

### 2.1. Materials

Polyvinyl alcohol, having a molecular weight of 66,000 g/mol (99% hydrolysed), was purchased from Siheung, Republic of Korea. Sodium sulfadiazine (NaSD) (Mw 272.26 g/mol), acetic acid, acetone, and ethanol were purchased from Sigma Aldrich (St. Louis, MO, USA). Polycaprolactone (Mw 80,000 g/mol) was purchased from Haihang Industry Co., Ltd. (Jinan, China). Deionized and distilled water were purchased from the local market.

### 2.2. Methods

#### Dope Solution Preparation and Fibre Development

**Figure 1:**
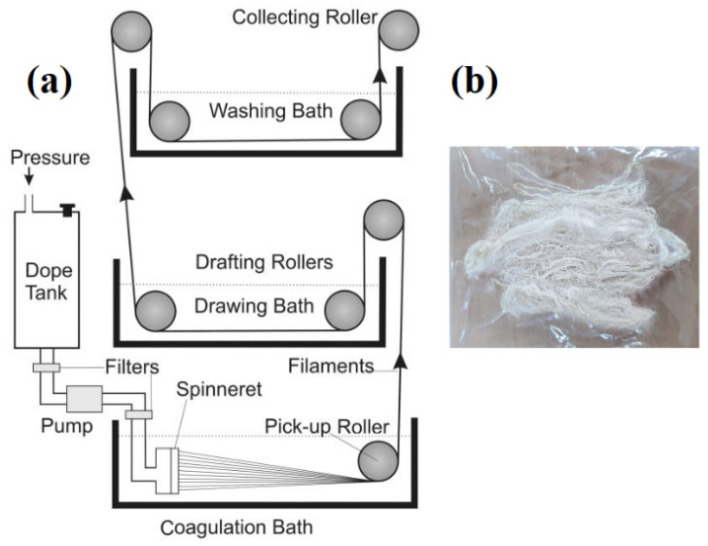
(**a**) Schematic illustration of the wet spinning technique for fibre production; (**b**) developed PVA/PCL fibres containing drug.

The dope solution of PVA and PCL, according to Table 1, was first prepared separately. PVA powder was dissolved in acetic acid and stirred at 800 rpm for 5 h at 90 °C until a homogenous mixture was obtained. Similarly, PCL pellets were also dissolved in acetic acid and stirred mechanically at 600 rpm for 6 h at 50 °C until a homogenous mixture was obtained. After that, both solutions were mixed and stirred to obtain a homogenous mixture. The model drug (NaSD), according to Table 2, was also inserted in this homogenous solution. This solution was poured into the dope tank of the wet spinning machine. The solution was kept overnight to remove air bubbles. The next day it was extruded at room temperature (22 °C ± 2 °C) via a spinneret with the help of a feeding pump at the speed of 5 rpm, the first roller speed at 8 rpm, and the second roller with the speed maintained at 20 rpm. The draw ratio was maintained at 2.2. Polymers dissolved in solvent were pushed through a spinneret (micron) and passed through the coagulation bath (first bath) comprising ethanol (100%) at 4 °C. The dope solution became solidified in this bath. In the second bath, fibres were rinsed (in deionized water), drawn, and collected on collecting rollers. The wet spinning setup is illustrated in Figure 1a. Fibres were dried after rinsing in water followed by immersion in acetone solution of different concentrations in 25%, 50%, 75%, and finally in 100% acetone. In each solution, fibres were kept for 30 min. Fibres in complete dry form were then stored in polythene zippers. Developed PVA/PCL fibres containing the drug are shown in Figure 1b.

### 2.3. Characterization

#### 2.3.1. Linear Density

According to ASTM D1059-12, 40 specimens each having a length of 2 inches were prepared from each sample and weighed on a digital weighing balance up to 4 decimals. Length was converted into meters. Ten measurements of each sample were taken and averaged. Then the linear density calculation was made by using Equation (1).

(1) $$Linear\;density\;\left(tex\right)=\frac{Weight\;in\;grams}{1000\;m}$$

#### 2.3.2. Tensile Properties

Tensile properties of the fibres were measured by using the tensile strength tester by using the standard ASTM D3822 method. A Testometric 2.5 single fibre strength tester was used. A sample was placed between 2 clamps, one fixed and one moveable, 10 mm apart. The force applied on the clamp was 10 N and set at 12 mm/min CRE (constant rate of extension). Five measurements of each sample were performed and averaged.

#### 2.3.3. Liquid Absorption

The absorption values of developed fibres were tested by using three different solutions, i.e., distilled water (DW), saline solution (SS) (0.9% *w*/*v* NaCl), and solution A (SA) (0.8298% *w*/*v* NaCl + 0.0368% *w*/*v* CaCl_2_). Developed fibres were soaked for 1 h in each solution and hung in the air to remove the excess liquid droplets and then weighed. Wet fibres were kept in the oven overnight at 105 °C to dry the fibres. Five measurements of each sample were made and averaged. By using Equation (2), liquid absorption was calculated.

(2) $$Absorption\;\left(\frac{g}{g}\right)=\frac{W_{w}-W_{d}}{W_{d}}$$

where *W_w_* and *W_d_* are the wet and dry weight of the fibre [60,61].

#### 2.3.4. Swelling Behaviour

An optical microscope attached with a digital camera was used (at 40X) to examine the swelling behaviour of developed fibre in three different above-mentioned solutions. Single fibre poured in these solutions was kept for 4 min at 25 °C, and then the diameter was measured. Five readings of each sample were taken and averaged. The swelling percentage was calculated by using Equation (3).

(3) $$Swelling\;\%=\frac{D_{w}-D_{d}}{D_{d}}\times 100$$

where *D_w_* and *D_d_* refer to wet and dry fibre diameter, respectively.

#### 2.3.5. Degradation Test

A degradation test was performed to measure the degradation % of the developed fibres. The PVA and PCL wet-spun fibres were then cut into specific lengths and dried in an oven and weighed (*W*_1_). The dried specimens were placed in capped bottles containing 250 mL of phosphate buffer solution (PBS, pH 7.4) with 0.3 mg hydrolase and then placed in a shaking incubator (100 rpm) at 30 °C for 24 h. After degradation time, specimens were removed and rinsed with distilled water thoroughly. Specimens were then dried in a desiccator and weighed (*W*_2_). The weight loss was calculated to assess the degradation rate by using Equation (4) [62]. Five readings of each sample were taken and averaged.

(4) $$Degradation\;\%=\frac{W_{1}-W_{2}}{W_{1}}\times 100$$

#### 2.3.6. FTIR

To study the chemical interaction between PVA, PCL, PVA/PCL, and drug-containing PVA/PCL fibres, Fourier transform infrared spectroscopy (FTIR) was performed. Infrared spectra of these fibres were measured by the attenuated total reflection (ATR) method using a Fourier transform infrared spectrometer connected to a PC with specific software analysis. All spectra were recorded between wave length 500 and 4000 cm^−1^.

#### 2.3.7. Scanning Electron Microscopy (SEM)

The morphology of PVA/PCL fibres containing the model drug was observed by using scanning electron microscopy (SEM Quanta 250). First, the specimen was brought into SEM holders and subsequently sputtered with gold. SEM uses incident electrons from an electron gun having a voltage of 10 kV under a high vacuum at a magnification range of 800x.

#### 2.3.8. Antimicrobial Efficiency

The standard testing method AATCC 147-1998 (American Association of Textile Chemists and Colorists) was used to check out the anti-microbial efficiency of developed fibres. An aliquot of approximately 25 μL of 10^−5^ dilution of the overnight incubated bacterial strains—*Staphylococcus aureus* (*S. aureus*) and *Escherichia coli* (*E. coli*)—was spread on the sterile agar plate. The specimens (drug-containing fibres) were placed on the agar surface and pressed gently to ensure intimate contact. The agar plates were then incubated at 37 °C for 24 h. After incubation, the agar plates were examined for microbial growth under and around the test specimens, and the zone of inhibition was observed. The affected zone was calculated by applying the formula given in Equation (5).

(5) $$W=\frac{T-D}{2}$$

In the above formula, ‘*W*’ stands for the width of the zone, which is clear from inhibition in millimetres, the total diameter of the test sample and the clear zone is denoted by ‘*T*’, and the diameter of the test sample is mentioned as ‘*D*’ in the formula.

#### 2.3.9. In Vitro Drug Release

**Figure 2:**
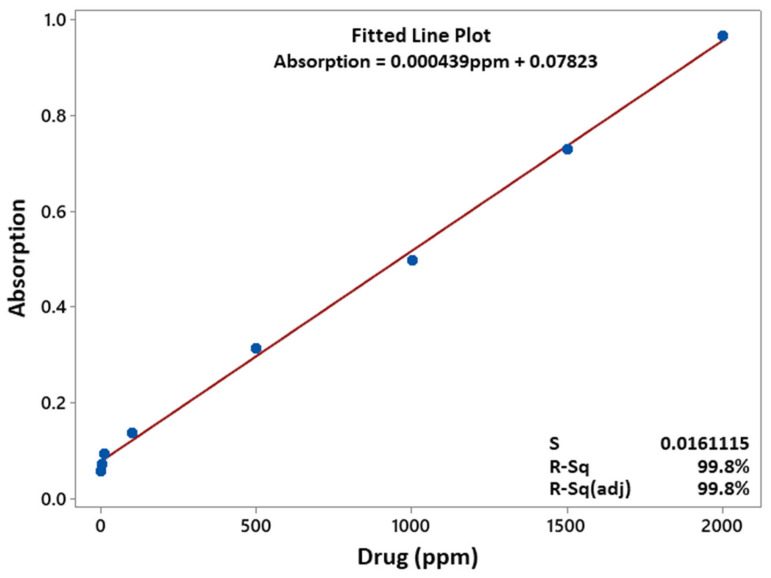
Linear regression equation and the standard curve.

To measure the quantity of released NaSD (drug) from the developed PVA/PCL fibres with the function of time, an in vitro study of the released drug was performed. A standard absorption curve at different concentrations of the drug was developed to measure the release of the drug. A series of drug loaded standard solutions of were prepared in distilled water to obtain a calibration curve. With a slit width of 5.0 nm, a Shimadzu UV–visible spectrophotometer (UV-2450) was used to measure absorption at 37 °C; 0.5 g of drug-loaded PVA/PCL fibres were placed into a 100 mL volumetric flask. This was filled with distilled water at 25 °C. Samples of 3 mL were drawn at 1, 2, 3, 8, 12, 24, 48, and 72 h (after mixing the solution with a micropipette). After each sampling, the solution was replenished with a similar solution of fresh dissolution media. Solutions were scanned in triplicate to measure the absorbance. A standard absorption curve at a maximum absorbance of 289 nm was drawn for the drug. From the standard absorption curve, a linear regression equation (fitted line plot) was developed. The linear regression equation and the standard absorption curve are shown in Figure 2.

## 3. Results and Discussion

### 3.1. Optimization of the Wet Spinning Process

Fibre surface characteristics are mainly dependent on its manufacturing process. In the preparation of wet spun fibres, different process parameters such as the non-solvent solution (coagulation solution), coagulation temperature, pump speed, and stretching and drawing rollers speed were optimized after many trials. Output was evaluated by linear density, SEM, tensile properties (tenacity and elongation), liquid absorption, swelling behaviour, degradation test, antimicrobial activity, and drug release profile test of the extruded fibres. Different concentrations of PVA and PCL (according to Table 1) were used. Acetic acid was used as a solvent for both PVA and PCL. Ethanol was used as a non-solvent solution in a coagulation bath at 4 °C (cold). A pump speed of 5 rpm and a draw ratio of 2.5 was used to obtain smooth and long fibres. All these optimized parameters of the wet spinning process to produce the fibres are given in Table 3.

### 3.2. Linear Density, Tenacity, and Elongation

**Figure 3:**
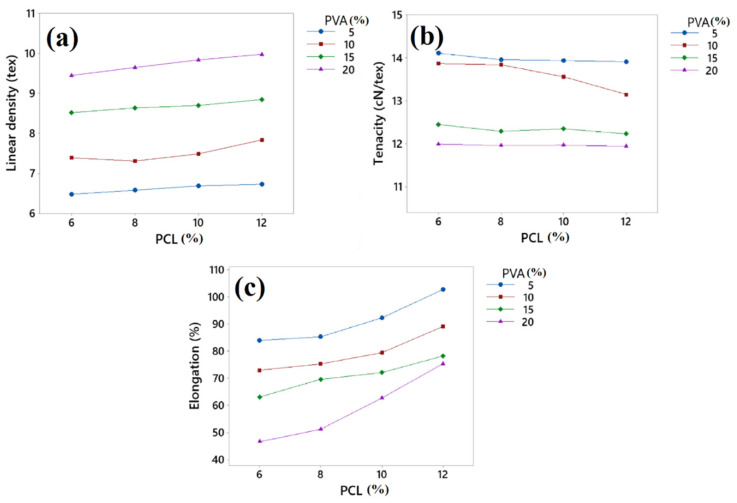
(**a**) Linear density; (**b**) tenacity; (**c**) elongation percentage of developed PVA/PCL fibres.

The linear density of the developed fibres was obtained in a range of 6.48 Tex to 9.98 Tex having an average value of 8.13 Tex. The comparative effect of PVA and PCL on the developed fibre linear density is shown in Figure 3a. From the results, it can be seen that both PVA and PCL have a direct effect on the developed fibres’ linear density. When the concentration of PVA increased, the fibres’ linear density also increased. The increase in fibre linear density might have been due to the increase in the viscosity of the solution, which is shown in Table 4. The addition of PCL also increased the fibre’s linear density, but the effect of PCL was less as compared to PVA. The concentration effect of PVA was more prominent than PCL concentration. This increase in linear density may have been due to the similar fact that the increase in PCL concentration resulted in an increase in viscosity, which resulted in an increased linear density to some extent.

The tenacity of the developed fibres was in the range of 14.11 to 11.94 cN/tex, as shown in Figure 3b. The tenacity of a fibre indicates the load-bearing capacity of the fibre per linear density of the fibre material. The higher the tenacity, the stronger will be the fibre. From the results, it can be seen that the PVA and PCL had an indirect effect on the tenacity of the developed fibres, as tenacity decreased by increasing the concentration of PVA and PCL. The developed fibre sample no. F16 (PVA/PCL 20/12 concentration) exhibited the lowest tenacity. This may have been due to the increase in viscosity because when the concentration of PVA and PCL increased, the viscosity of the solution also increased, which resulted in increases in the linear density. The reason might have been due to the irregular arrangement of polymeric chains by the increase in viscosity of the solution, as the polymeric chains might not have had sufficient time to arrange themselves in a regular manner with an increase in solution viscosity, hence reducing the load bearing capacity of the fibre.

Elongation % is the elasticity of a fibre. It indicates the limit of fibre extension without any breakage. To determine the mechanical properties of the developed fibres, elongation was used. A higher elongation % is beneficial when the fibres have to face external forces. Elongation of the developed fibres was in the range of 102.73% to 46.61%. Figure 3c represents the effect of PVA and PCL on the elongation % of the developed fibres. It was found that PVA had a negative effect on the elongation % of developed fibres. By increasing the concentration of PVA, elongation % decreased. This has maybe due to the crystalline structure of PVA. The regular placement of polymeric chains reduced the elongation %, while on the other hand, PCL had a positive effect on the elongation % of the developed fibres. When the concentration of PCL increased, elongation also increased to a significant level. This may have been due to the elastomeric nature of PCL. Furthermore, an increase in PCL concentration resulted in a reduced crystalline behaviour of developed fibres, which also increased the elongation % [63].

### 3.3. Liquid Absorption (g/g)

**Figure 4:**
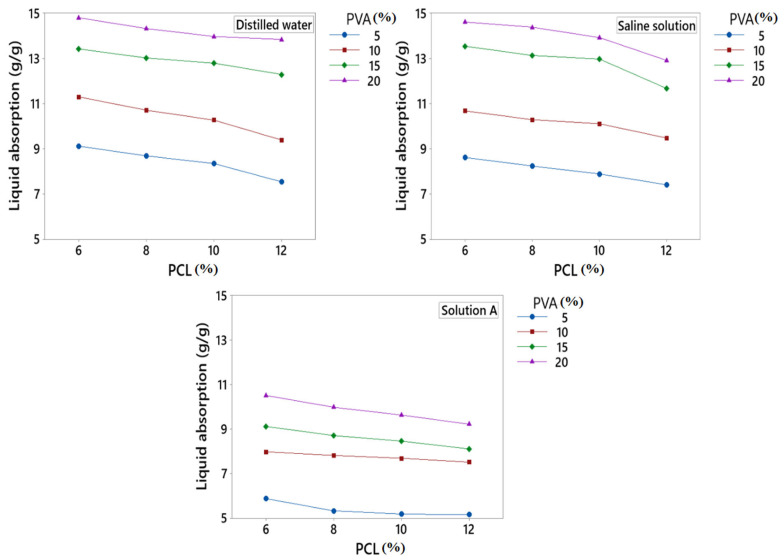
Liquid absorption of developed PVA/PCL fibres.

The absorption (g/g) of the developed fibres was tested in three different solutions, i.e., DW, SS, and SA, as shown in Figure 4. It was observed from the results that fibres showed higher absorption in DW. This may have been due to the easy accessibility of water molecules to highly absorbent fibres. Moreover, hydroxyl groups in the PVA enhanced the fibre’s water absorption properties. As compared to DW, fibres showed less absorption properties in SS due to the presence of Na^+^ ions, which reduced the ion exchange and resulted in less absorption. In comparison to SS and DW, the novel fibres exhibited very low absorption (g/g) in SA; this may have been due to the presence of calcium ions in SA, which created hindrance in ion-exchange, which resulted in less absorption.

Liquid absorption (g/g) of the fibres increases by increasing the concentration of PVA. When the concentration of PVA increases, OH groups also increase, which attracts the liquid molecules and results in increased liquid absorption. PCL has an indirect impact on the absorption of developed fibres because PCL is hydrophobic, so when the concentration of PCL increases, liquid absorption decreases at a constant concentration of PVA.

### 3.4. Swelling %

**Figure 5:**
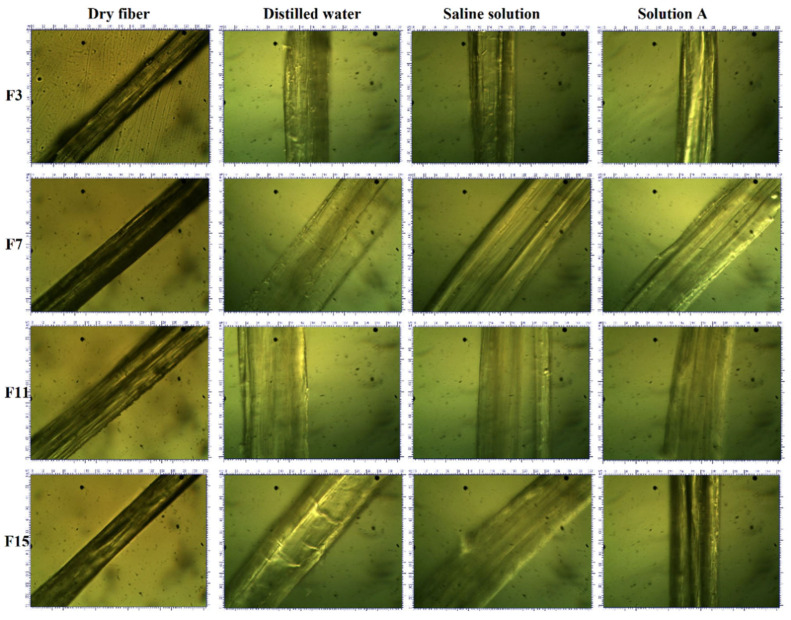
Comparison of swelling behaviour of PVA/PCL fibres at 40X.

For an ideal wound dressing, excessive exudate management is a very important thing, which is usually examined by the swelling % of the fibres. The swelling % of developed fibres was analysed and calculated in three different above-mentioned solutions by using an optical microscope attached with a digital camera at 40X, which is shown in Figure 5.

**Figure 6:**
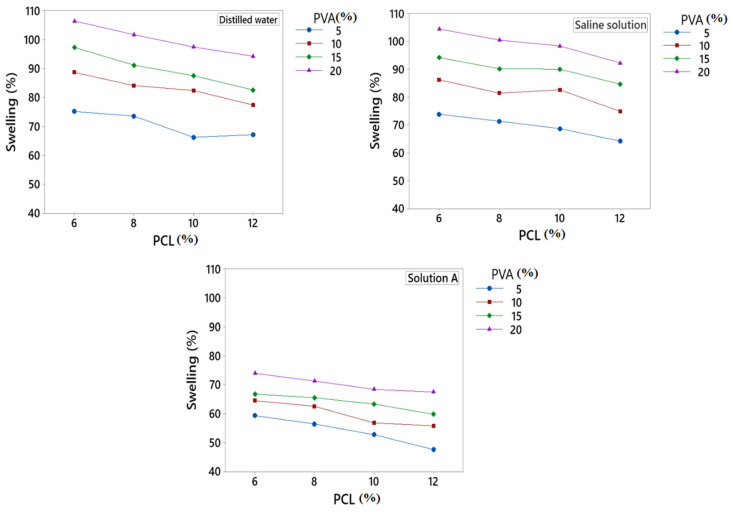
Swelling % of developed PVA/PCL fibres.

Figure 6 shows the fibre swelling % in three different solutions. From the results, it was observed that the developed fibres had the highest swelling % in DW due to the presence of hydroxyl groups in PVA. The presence of OH groups in PVA makes it a good hydrophilic polymer [64]. Therefore, when the concentration of PVA increases, the number of OH groups increase, which attracts water molecules to make the fibres swell more. The increase in linear density may also be due to the non-presence of sodium and calcium ions in water. Developed fibres showed less swelling % in SS as compared to DW. The presence of sodium ions in SS reduced the ion exchange, which resulted in less swelling %. SA had the lowest swelling % as compared to DW and SS. In SA, the Ca^+^ ion reduced the swelling % due to the creation of hindrance in ion exchange. The hindrance of ions in SA resulted in decreased swelling %.

The concentration of PVA and PCL also affected the swelling % of the developed fibres. When the concentration of PVA increased, the swelling % increased due to the hydrophilic nature of PVA. Moreover, with the increase in PVA concentration, hydroxyl groups increased, which resulted in increased swelling % of the developed fibres. On the other hand, PCL had a negative effect on the swelling % of the developed fibres. When the concentration of PCL increased, the swelling % decreased due to the hydrophobic nature of PCL. Overall swelling % increased by increasing the PVA at a constant PCL concentration.

### 3.5. Degradation Test

**Figure 7:**
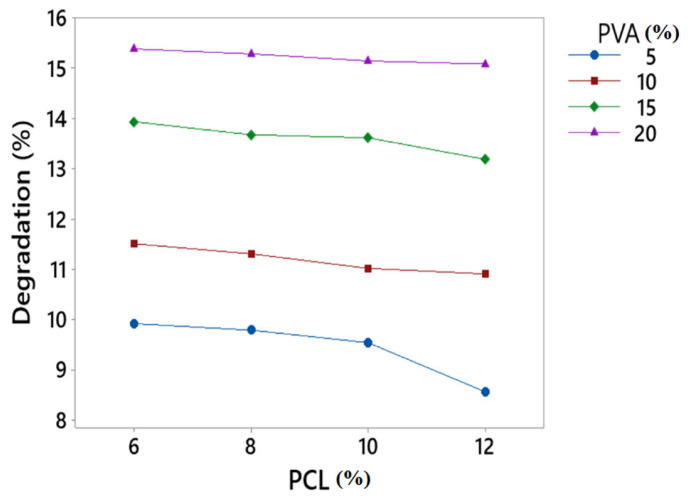
Degradation of developed PVA/PCL fibres.

The degradation test was performed to measure the degradation of developed fibres. Figure 7 shows the degradation rate of PVA/PCL fibres. From the results, it was observed that PVA had a direct impact on the degradation of the developed fibres. By increasing the concentration of PVA, the degradation rate increased. PVA is highly soluble in water and has the ability to reduce weight when dissolved in liquid media for many hours. Therefore, when the concentration of PVA increased, the degradation % of the developed fibres also increased. On the other hand, PCL had less effect on degradation % as compared to PVA. When the concentration of PCL increased, degradation increased but to some extent because PCL degraded slowly as compared to PVA.

### 3.6. FTIR

**Figure 8:**
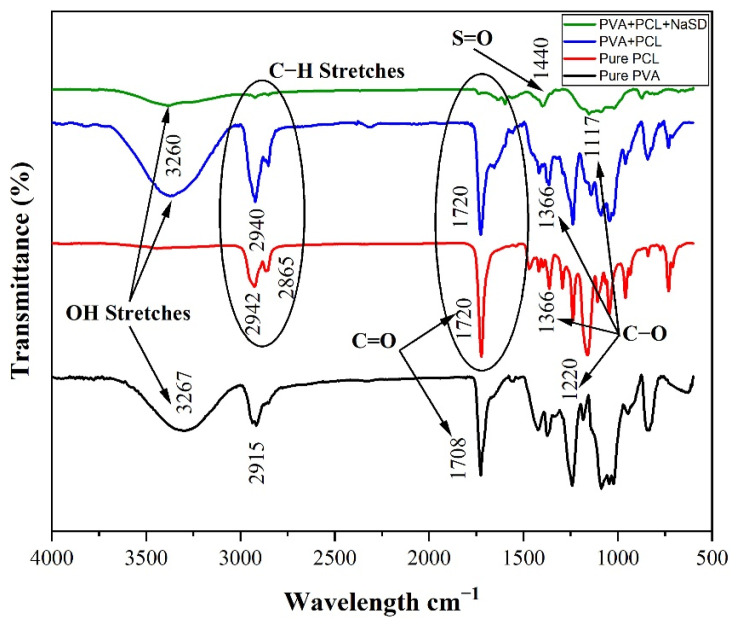
FTIR spectra of PVA, PCL, PVA/PCL, and drug-containing PVA/PCL fibres.

The FTIR spectra of PVA, PCL, PVA/PCL, and drug-containing PVA/PCL fibres were performed to identify the functional groups after the successful incorporation of PVA in PCL as well as drug in PVA/PCL solution. Figure 8 shows the FTIR spectra of PVA, PCL, PVA/PCL, and drug-containing PVA/PCL fibres. Confirmation of different materials used in the development of fibres was carried out by the detection of functional groups having specific peak areas. The characteristics peaks for PVA were depicted at 3267 cm^−1^, 2915 cm^−1^, 1708 cm^−1^, and 1220 cm^−1^, which confirmed the presence of O–H stretching, C–H stretching, carbonyl group (C=O) stretching, and ester group stretching, respectively [53]. In the case of PCL, characteristics peaks were depicted at 2942 cm^−1^, 2865 cm^−1^, 1720 cm^−1^, and 1366 cm^−1^, showing the presence of CH_2_-asymmetric stretching, CH_2_ symmetric stretching, carbonyl group stretching, and C–H_2_ bending vibrations, respectively [53]. A blend of PVA/PCL fibres showed peaks at 3260 cm^−1^, 2940 cm^−1^, 1720 cm^−1^, and 1117 cm^−1^, which confirmed the presence of O–H stretching, C–H_2_ stretching, and C=O carbonyl group stretching and C–H_2_ bending vibrations, respectively. The characteristic peaks of PVA and PCL in a blend of PVA/PCL fibre confirmed the successful co-extrusion of the two polymers. The peak depicted at 1117 cm^−1^ confirmed the presence of the C–O ester group in PCL. In the developed PVA/PCL fibre-containing drug, the peak at 1440 cm^−1^ was related to the asymmetric stretching vibration of the S=O sulphite functional group, which confirmed the presence of NaSD. The FTIR spectra confirmed the successful loading of NaSD (drug) in the developed PVA/PCL fibres.

### 3.7. Scanning Electron Microscopy

**Figure 9:**
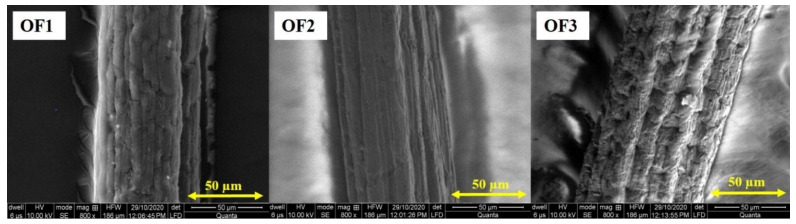
Surface morphologies of drug-loaded fibres OF1, OF2, and OF3 containing 1%, 1.5%, and 2% drug.

The developed fibres were subjected using scanning electron microscopy to analyse their surface and porosity. Three optimized fibres were selected based on varying drug concentrations, which are given in Table 2, to check and verify the effect of drug concentration on the surface morphology of the developed fibres. From Figure 9, it is observed that with the change in drug concentration, the surface roughness increased. A smooth surface was obtained for the minimal drug concentration, which changed into a rough surface with an increase in drug concentration in the fibre. Slashes on the surface of the developed fibres could be observed in the longitudinal direction. This may have been due to the extrusion process of the developed fibres because the dope solution was extruded through a spinneret having circular holes directly into a coagulation bath containing ethanol (at 4 °C), which solidified the fibres quickly. The rough spinneret surface may have caused roughness in fibres after the coagulation process. This also supported a higher surface area, which facilitated better drug release per cross-sectional area. Images of developed optimized fibres containing drug in the longitudinal view were taken, which are shown in Figure 9.

### 3.8. Antimicrobial Activity

**Figure 10:**
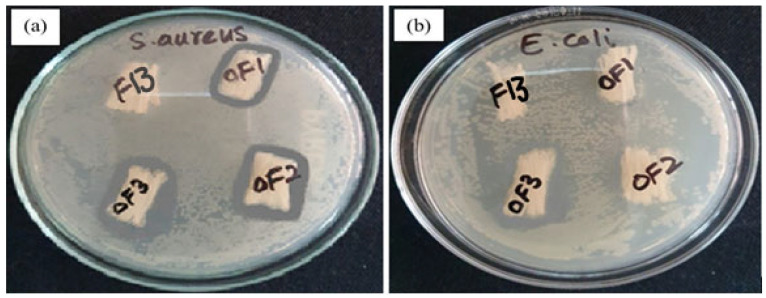
Antimicrobial activity of drug-loaded optimized fibres against (**a**) *Staphylococcus* and (**b**) *Escherichia coli*.

To analyse the relative effect of the drug, antimicrobial activity was performed for the optimized fibre with three different concentrations of the drug according to Table 2. Antimicrobial activity was tested against two different bacterial strains, i.e., *Staphylococcus aureus* (Gram-positive), the most commonly studied in wound practices, and *Escherichia coli* (Gram-negative). Figure 10 shows the zone of inhibition resulting from the developed OF1, OF2, and OF3 fibres containing 1%, 1.5%, and 2% drug, respectively. It is clearly seen from the photographs of agar discs that the optimized fibres were effective against *S. aureus* bacterial strains and showed no effect against *E. coli* bacterial strains. This may have been due to differences in their bacterial cell wall structures. Gram-negative bacteria have an outer cell membrane, which is not found in Gram-positive bacteria. Gram-positive bacteria’s cell wall is rich in peptidoglycan, which is responsible for preserving the Gram-staining dye and iodine solutions/liquids of Gram [41,65,66]. Table 5 shows the zone of inhibition against the *S. aureus* bacterium. Twenty readings of each sample were taken from the fixed centre of samples to different edges of fibres and zones of inhibition as well. The average radius of fibres and zone of inhibition was calculated from these readings. Through this radius, the average diameter of fibres and zone of inhibition was calculated.

From Figure 10a, it is observed that the zone of inhibition against *S. aureus* bacterial strains was at its maximum at a 2% drug concentration and a minimum at 1% drug concentration, which showed the presence of the drug in the developed fibres, while no bacterial strains were found in the F13 sample, which was without a drug. OF1, OF2, and OF3 fibres showed a clear inhibition zone with a particular diameter, while the fibres without drug showed bacterial colonies. It may be considered that the drug-containing fibres exhibiting a higher release of the drug showed a bigger inhibition zone as compared to those without drug fibres (F13). As the developed fibres containing the drug exhibited good antibacterial activity against *S. aureus* that causes wound infections, the developed fibres can be used for preventing infection if used as a dressing either alone or with some supportive dressings.

### 3.9. In Vitro Drug Release

**Figure 11:**
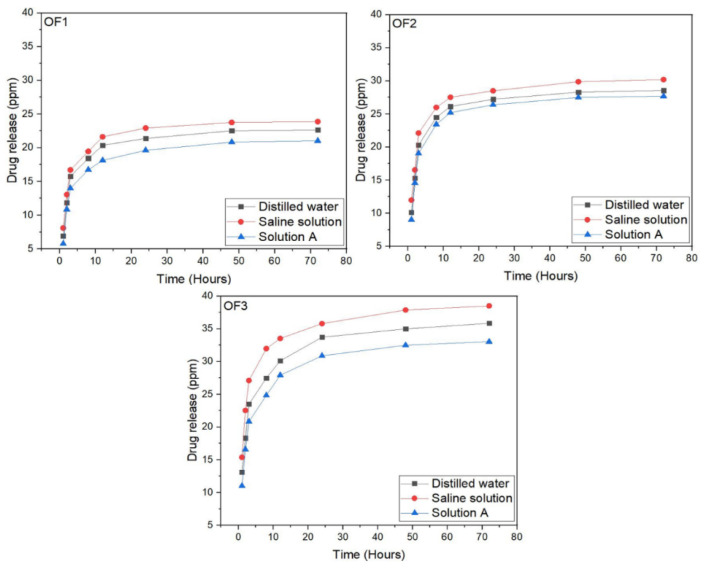
Drug release profile of developed optimized PVA/PCL fibres.

Figure 11 shows the controlled release of the drug (NaSD) from OF1, OF2, and OF3 fibres at different time intervals. From the results, it was seen that the developed optimized fibres showed a maximum release of the drug in the first three hours. This may have been due to the burst release of the drug. The process by which the drug is released involves the dissolution of the drug followed by diffusion to enter the release medium via the swelling grooved structure, and this entire mechanism proceeds at the interphase. The experimentally measured initial rapid release of the drug is known as the burst effect, which occurs due to the large interphase (due to rough fibres’ surfaces) between developed fibres and dissolution medium, which contributes to the dissolution and migration of the drug onto the fibres’ surface and results in the burst release of drug in the first three hours. The slow-release rate of the drug is reached after this burst release of the drug. The non-covalent interaction between the hydrophilic polymer (PVA) and drug (dispersed in fibres’ rough structures) is greatly increased as a consequence of the large surface area. This phenomenon limits the drug’s diffusion and thus contributes to a drop in the rate of drug release.

From Figure 11, it is also observed that the drug showed its maximum release in SS. SS has a high amount of sodium ions, which may have resulted in the production of water-soluble sodium ions and caused the higher release of the drug. The release of the drug was better in DW as compared to SA. However, this release was less than in SS. The higher release of the drug in DW may be due to the easy accessibility of water molecules to the drug. However, the release of drug in DW is only due to its soluble nature. In the case of SA, the presence of Ca^+^ in the solution resulted in the lowest release of the drug. Calcium ions in SA hinder ion exchange, which may result in less release of the drug.

## 4. Conclusions

In this study, hydrophilic PVA/PCL fibres containing a model drug were prepared by using the wet spinning technique. Process parameters were set after initial trials. The novel developed hydrophilic fibres were used as a substrate to load drug for wound dressing applications. From the results, it is concluded that developed fibres can be produced by using PVA and PCL as a blend containing the drug by passing through a coagulation bath containing ethanol at 4 °C. The inclusion of PVA and PCL increases the linear density. Absorption (g/g) and swelling % of the developed fibres increase to a significant level by increasing the PVA concentration but decrease by increasing the PCL concentration. The degradation % of the developed fibres also depends on the PVA concentration. As PVA content increases, the degradation % of the developed fibres also increases. Degradation % decreases when the PCL concentration increases at a constant PVA. FTIR confirmed the presence of the drug in the developed fibres. Developed fibres also have good resistance against *S. aureus* bacteria with good drug release. Good mechanical properties, absorption (g/g), swelling %, degradation %, and enhanced antimicrobial activity with an appropriate drug release profile test nominate the developed PVA/PCL fibres containing the model drug as a promising wound dressing candidate for wound dressing applications.

## Figures and Tables

**Table 1:** Design of experiment.

Sr. No.	PVA Concentration % (*w*/*v*)	PCL Concentration % (*w*/*v*)	Sample Code
1	5	6	F1
2		8	F2
3		10	F3
4		12	F4
5	10	6	F5
6		8	F6
7		10	F7
8		12	F8
9	15	6	F9
10		8	F10
11		10	F11
12		12	F12
13	20	6	F13
14		8	F14
15		10	F15
16		12	F16

**Table 2:** Incorporation of model drug in the optimized fibre blend.

Sr. No.	PVA/PCL Conc. % (*w*/*v*)	NaSD % (*w*/*v*)	Drug Containing Fibres
1	PVA 20 + PCL 6	1	OF1
2	PVA 20 + PCL 6	1.5	OF2
3	PVA 20 + PCL 6	2	OF3

**Table 3:** Optimization parameters of wet spinning setup.

Sr. No.	Spinning Parameters		
		Bath 1	Bath 2
1	Spinning bath	100% Ethanol	Deionized water
2	Temperature (°C)	4	25
3	Speed of rollers (rpm)	8	20
4	Pump speed (rpm)	5	

**Table 4:** Viscosity of PVA/PCL solutions.

Sample Type	PVA/PCL Conc. % (*w*/*v*)	Viscosity (Pa.s)
F7	PVA10 + PCL 10	2.52
F10	PVA15 + PCL 8	8.56
F11	PVA15 + PCL 10	10.61
F12	PVA15 + PCL 12	13.99
F15	PVA20 + PCL 10	24.99

**Table 5:** Antimicrobial activity (zone of inhibition) results.

Sample Type	Zone of Inhibition (mm)
	*S. aureus*
F13 (without drug)	Not formed
OF1	5.33
OF2	7.57
OF3	8.29

## Data Availability

Data will be available upon request.
